# The Effect of Inactivated SARS-CoV-2 Vaccines on TRAB in Graves’ Disease

**DOI:** 10.3389/fendo.2022.835880

**Published:** 2022-05-16

**Authors:** LingHong Huang, ZhengRong Jiang, JingXiong Zhou, YuPing Chen, HuiBin Huang

**Affiliations:** ^1^ The Second Clinical Medical College of Fujian Medical University, Quanzhou, China; ^2^ Department of Endocrinology, The Second Affiliated Hospital of Fujian Medical University, Quanzhou, China

**Keywords:** inactivated SARS-CoV-2 vaccine, Graves’ disease (GD), thyrotropin receptor antibody (TRAB), autoimmune disease (AID), autoimmune thyroid disease (AITD)

## Abstract

**Background:**

The ongoing coronavirus disease 2019 (COVID-19) pandemic has forced the development of vaccines. Reports have suggested that vaccines play a role in inducing autoimmune diseases (AIDs). Scattered cases have reported that severe acute respiratory syndrome coronavirus 2 (SARS-CoV-2) vaccines may promote thyroid disease, including Graves’ disease (GD). However, the effect of inactivated SARS-CoV-2 vaccine on GD remains unclear. The aim of the present study was to investigate the response of thyrotropin receptor antibody (TRAB) to inactivated SARS-COV-2 vaccines.

**Methods:**

We conducted a retrospective study to observe the differences in thyroid function and TRAB trends between pre-vaccination (n=412) and post-vaccination (n=231) groups at an interval of 2 months. We then retrospectively observed the differences in serum thyroid function and TRAB levels at 3 months before (n=280), 1 month before (n=294), 1 month after (n=306), and 3 months after (n=250) vaccination. Subsequently, 173 GD patients who were not vaccinated with inactivated SARS-COV-2 vaccines were selected for a prospective study. Thyroid function and TRAB assessment were performed before 3 and 1 months and 1 and 3 months after the first dose of vaccination and were then compared by repeated measures ANOVA to explore their dynamic changes.

**Results:**

A retrospective study preliminarily observed that the trend of TRAB post-vaccination was opposite of that pre-vaccination (p=0.000), serum TRAB levels decreased before vaccination and increased after vaccination. In this prospective study, repeated measures ANOVA indicated significant differences in serum FT3 (p=0.000), FT4 (p=0.000), TSH (p=0.000), and TRAB (p=0.000) levels at different time points before and after vaccination. Serum TRAB levels showed dynamic changes that decreased significantly at 1 month before vaccination (p=0.000), no significant differences at 1 month after vaccination (p=0.583), and reflected an upward trend at 3 months after vaccination (p=0.034). Serum FT3 and FT4 levels showed similar trends to serum TRAB levels before and after vaccination. Instead, the serum TSH levels showed a continuous upward trend over time.

**Conclusion:**

Based on the results obtained in both retrospective and prospective studies, we concluded that serum TRAB levels decreased less after inactivated SARS-CoV-2 vaccination and showed an upward trend, which may be related to humoral immunity induced by vaccination.

## Background

The coronavirus disease 2019 (COVID-19) pandemic, caused by severe acute respiratory syndrome coronavirus 2 (SARS-CoV-2), has infected more than 20 million individuals and caused more than 5 million deaths worldwide by December 12, 2021. COVID-19 can cause both pulmonary and systemic inflammation, potentially leading to multi-organ dysfunction. Thyroid diseases, including thyrotoxicosis, hypothyroidism, and non-thyroid disease syndromes, can also be caused by COVID-19 (1). To date, there is no specific treatment for SARS-CoV-2, and vaccination is a basic and effective way to prevent the spread of this virus. Currently, the types of vaccines in use include inactivated virus vaccines, live attenuated virus vaccines, nucleic acid vaccines, recombinant viral vector vaccines, and recombinant subunit vaccines (2). In China, more than 1 billion people have been vaccinated with inactivated SARS-CoV-2, and mass vaccination continues.

Graves’ disease (GD) is an organ-specific autoimmune disease (AID) which is characterized by thyrotropin receptor antibody (TRAB). Genetic factors account for 80% of the risk of developing GD, whereas the other 20% are related to environmental risk factors (3). For example, autoimmune thyroid disease (AITD) is a common side effect of alemtuzumab therapy in patients with multiple sclerosis (4). These factors contribute to the onset of GD in genetically susceptible individuals by breaking down the mechanisms that lead to immune tolerance. The immunopathogenesis of GD is complex, and TRAB is the ultimate cause of hyperthyroidism (5). It binds to the thyroid-stimulating hormone (TSH) receptor on the surface of thyroid follicular cells, resulting in persistent and uncontrolled thyroid stimulation, leading to abnormal overproduction of thyroid hormones and hyperthyroidism.

Vaccines have long been suspected to play a role in inducing AIDs (6). There have been isolated case reports of arthritis, vasculitis, and central or peripheral nervous system symptoms following vaccination. Although these cases tend to be very infrequent, there have been reports of AID after SARS-CoV-2 vaccination, including AITD. SARS-CoV-2 vaccines-induced thyroid disease is not a single report. Recently, Alberto et al. and Zettinig et al. successively reported four cases of GD with positive TRAB induced by SARS-Cov2 RNA vaccination, which met the diagnostic criteria for autoimmune/inflammatory syndrome induced by adjuvants (ASIA) (7, 8). Another study reported that three women developed anterior neck pain after inactivated SARS-CoV-2 vaccine and were diagnosed with subacute thyroiditis, which is also thought to be a phenomenon of ASIA (9). Furthermore, one woman with a history of controlled GD developed ocular symptoms and signs after the mRNA COVID-19 vaccine, which, combined with elevated thyroid-stimulating immunoglobulin and orbital imaging, was consistent with a diagnosis of active Graves ophthalmopathy (GO) (10). Among the outpatients in our hospital, there were also cases of AITD induced by inactivated SARS-CoV-2 vaccines, including GD and Hashimoto’s thyroiditis (HT), which mainly manifested as hyperthyroidism, such as a swollen neck, palpitations, and weight loss (this case report is in the process of publication). In the aforementioned reports, there were both newly diagnosed and recurrent or aggravated cases.

Until now, little was known about the effect of inactivated SARS-CoV-2 vaccines on GD. As a marker of diagnosis and evaluation of treatment and remission, TRAB was the entry point of the present study. This study aimed to explore the response of TRABs in GD after inactivated SARS-CoV-2 vaccines in retrospective and prospective studies to further investigate the factors that may modulate these responses.

## Subjects and Methods

### Study Population

**Figure 1:**
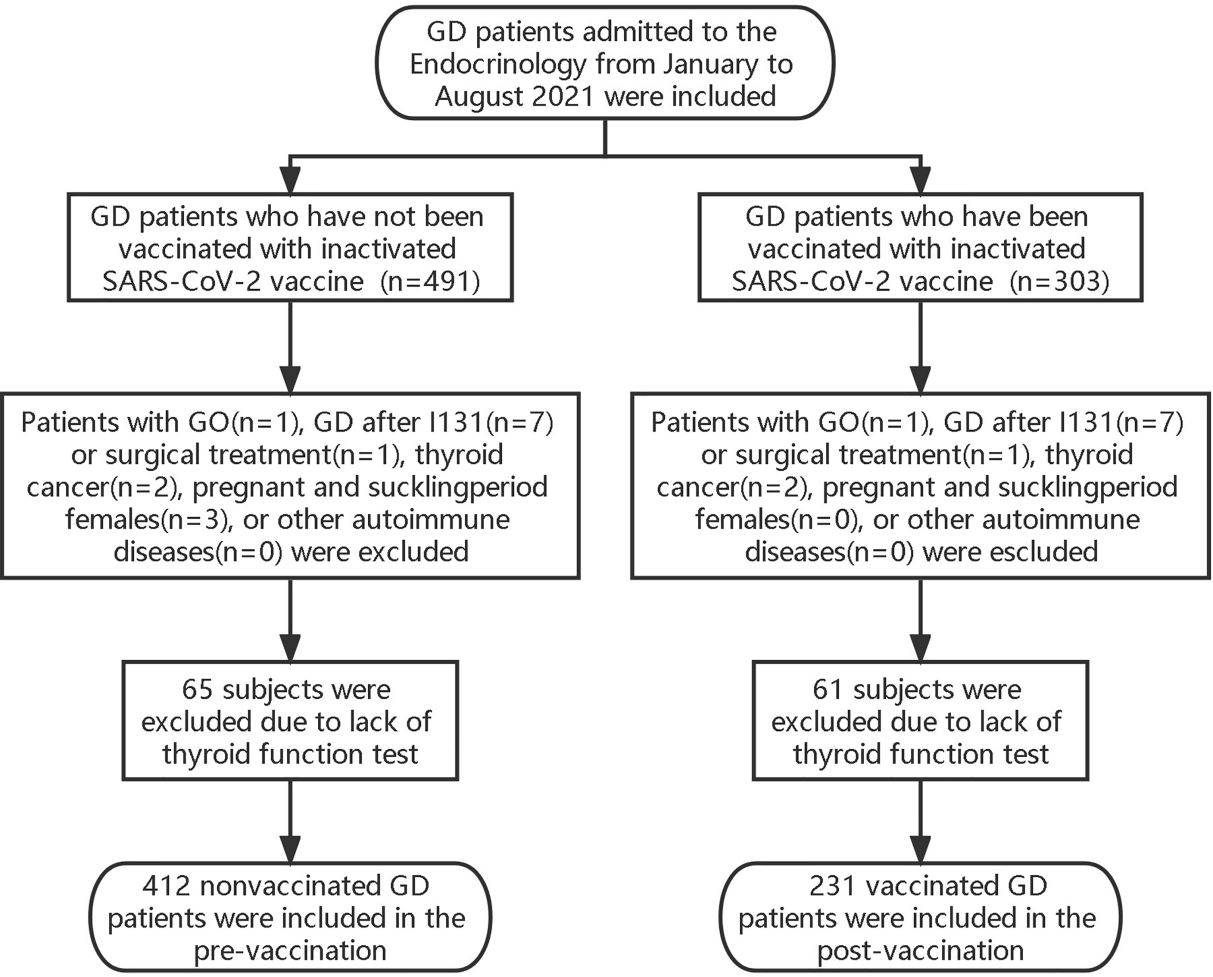
Flow charts of retrospective study population.

These retrospective studies were performed to observe the effects of vaccination on thyroid function before and after vaccination. We included all GD patients in the Endocrinology Department of the Second Affiliated Hospital of Fujian Medical University from January to August 2021. The patients included conformed to the diagnostic and treatment criteria of GD of the European Thyroid Association (ETA) (2018). Patients with GO, GD after I131 or surgical treatment, thyroid cancer, pregnant and suckling period females, or other autoimmune diseases were excluded (**Figure 1**). All enrolled patients were treated with methimazole (MMI) combined with levothyroxine (L-T4) to avoid drug-induced hypothyroidism according to the ATD, which is the first-line treatment for GD. All of them were biochemically consistent with hyperthyroidism and were TRAB-positive, and treatment regimens remained unchanged during the study period. According to the time point of the first vaccination, the included population was divided into pre-vaccination (n=412) and post-vaccination (n=231) groups. Pre-vaccination referred to patients with GD who had not yet been vaccinated with inactivated SARS-CoV-2 vaccine. Post-vaccination, the GD was vaccinated with inactivated SARS-CoV-2 vaccine. We collected data on thyroid function and TRAB levels, which were measured at 2-month intervals within a specified time through the clinical system.

We then retrospectively observed the differences in serum thyroid function and TRAB levels at different times before and after vaccination. We included all GD patients who received the first dose of inactivated SARS-CoV-2 vaccine in the Endocrinology Department of the Second Affiliated Hospital of Fujian Medical University from January to October 2021 (n=482). Patients with GO (n=1), GD after I131 (n=8) or surgical treatment (n=1), thyroid cancer (n=2), pregnant and suckling period females (n=0), or other autoimmune diseases (n=0) were excluded. Thyroid function and TRAB levels of the included GD patients were reviewed 3 months before, 1 month before, 1 month after, and 3 months after vaccination according to the time point of the first vaccination. After excluding patients with missing checklists at all the above time points (n=156), 314 patients were included and divided into four groups according to time points: pre-vaccination -3 month and -1 month and post-vaccination +1 month and +3 months. Due to the absence of follow-up at some time points, the cases in each group were as follows: pre-vaccination -3 months (n=280) and -1 month (n=294) and post-vaccination +1 month (n=306) and +3 months (n=250).

Subsequently, we conducted a prospective study on GD patients who were admitted to the Endocrinology Department of our hospital between March and May 2021 and had not been vaccinated. The enrolled population also met the diagnostic and treatment criteria of GD in ETA (2018), and the treatment regimen followed first-line treatment, which remained unchanged during the study period. According to their willingness and the control of hyperthyroidism, they are advised to receive inactivated SARS-COV-2 vaccines. The exclusion criteria were as follows: 1. GO; 2. patients who needed to change treatment due to illness during the test; 3. GD after I131 or surgical treatment; 4. thyroid cancer; 5. pregnant and suckling period females; 6. GD accompanied by serious medical diseases, liver and kidney dysfunction, or granulocytopenia; and 7. other autoimmune diseases. Finally, we enrolled 173 GD patients who had not received inactivated SARS-COV-2 vaccines. All subjects provided informed consent to participate in the study, which was approved by the local ethical committee.

### Vaccination

We reviewed the vaccination information in the Fujian Health Code to confirm and collect the type and date of vaccination and vaccine manufacturers of the participants to ensure the accuracy of information collection. They were also asked about their discomfort after the vaccination. The vaccine manufacturers of the enrolled patients included SINOVAC, Beijing Bio, and Chengdu Bio, which produced inactivated SARS-COV-2 vaccines.

### Assays

Venous blood samples were collected before 3 and 1 months and 1 and 3 months after the first dose of inactivated SARS-COV-2 vaccines. Serum free triiodothyronine 3 (FT3), free thyroxine 4 (FT4), thyroid-stimulating hormone (TSH), and TRAB levels were measured using a competitive electrochemiluminescence immunoassay (ECLIA) according to the manufacturer’s instructions (Roche COBAS-E601). Normal ranges of these parameters were as follows: FT3 (3.1-6.8pmol/L), FT4 (12.0-22.0pmol/L), TSH (0.27-4.20mIU/L), TRAB (0.00-1.75IU/L). The samples were analyzed in routine clinical laboratories at the Second Affiliated Hospital of Fujian Medical University.

### Statistical Analysis

All analyses were performed using Statistical Package for the Social Sciences software version 23 (SPSS Inc., Chicago, IL). Continuous variables were normally distributed and shown as mean ± standard deviation (x̅ ± s) and irregularly distributed data were expressed as medium (interquartile range). The Kolmogorov-Smirnov test was used for variables with skewed distributions. We used Blom’s formula to transform the skewed distribution into a normal distribution. The paired t-test and *post hoc* one-way analysis of variance (ANOVA) were used to assess the statistical significance of differences among the groups. Repeated measures ANOVA was performed to compare dynamic changes in thyroid function and TRAB levels in this prospective study. Statistical significance was set at p < 0.05.

## Results

### The Change Trend of TRAB in Post-Vaccination Was Opposite of That in Pre-Vaccination

**Figure 2:**
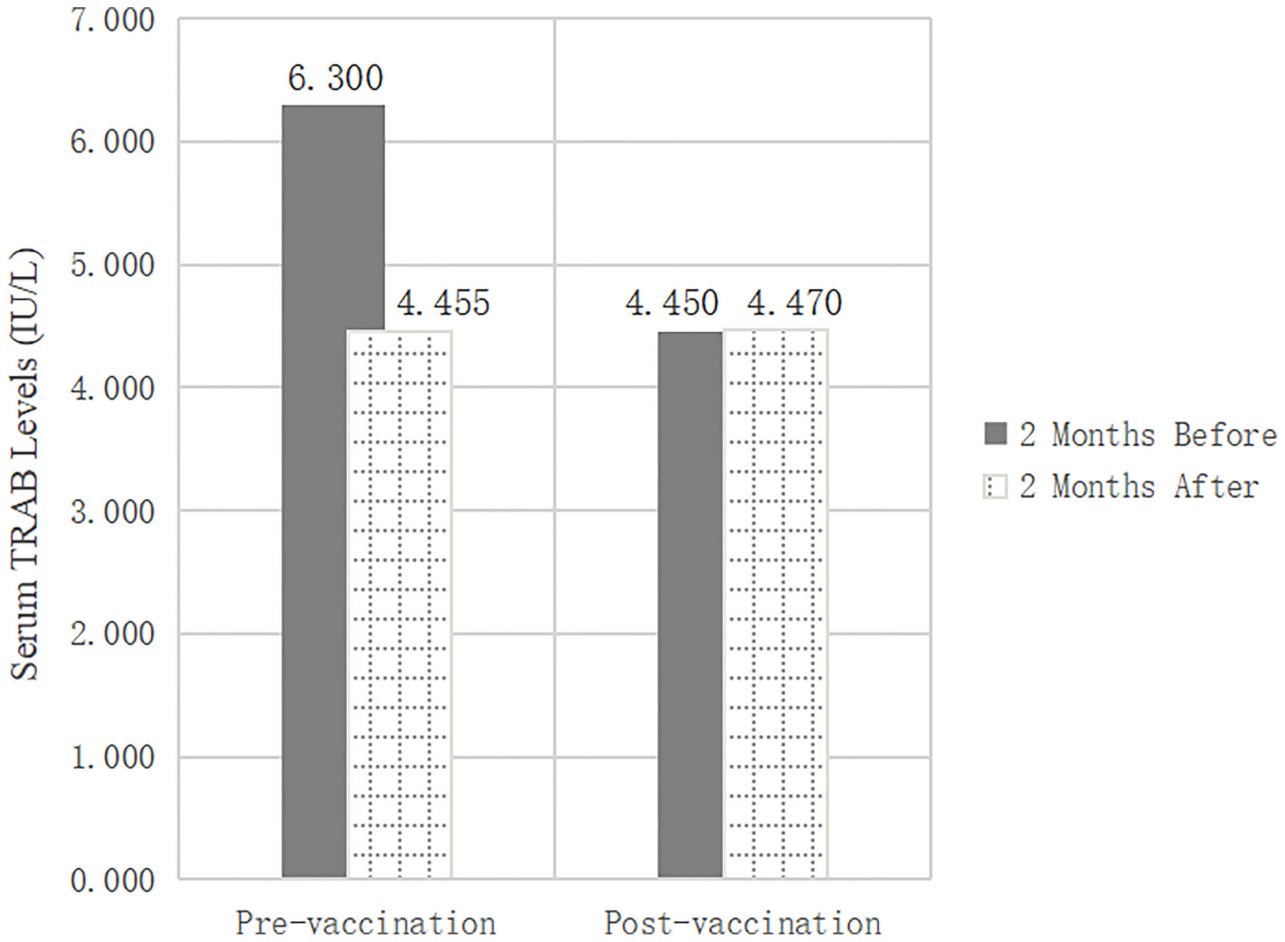
Changes of TRAB levels and amplitude between pre-vaccination and post-vaccination groups.

In this retrospective study, we reviewed changes in thyroid function and TRAB in populations at different stages of vaccination, including pre-vaccination (n=412) and post-vaccination (n=231), to determine the effect of inactivated SARS-CoV-2 vaccines on TRAB. The baseline clinical data of the two groups were analyzed statistically to exclude other influencing factors after vaccination for GD. No significant differences were observed between the groups in terms of sex, age, medication, or other clinical characteristics. Serum FT3 (p=0.000), FT4 (p=0.000), and TRAB (p=0.000) levels were significantly lower after 2 months than before pre-vaccination. There were no differences in thyroid function and TRAB between the 2-month post-vaccination intervals. The t-test showed that the TRAB change was statistically different between the two groups (p=0.000). In contrast to the pre-vaccination values, the TRAB change trend post-vaccination (1.290IU/L vs. 0.060IU/L) (p=0.000) was the opposite (**Table 1** and **Figure 2**).

**Table 1:** Comparison of clinical characteristics and thyroid function between groups.

	Pre-vaccination (n = 412)			Post-vaccination (n = 231)			P
Gender (female%)	77.910			76.190			0.618
Age (year)	39.020 ± 11.298			38.830 ± 10.895			0.941
Duration (month)	12.067 (5.433,20.725)			15.167 (9.300,25.600)			0.000
Thiamazole (mg)	15.127± 4.902			14.946 ± 5.563			0.844
Letrox (ug)	54.854 ± 36.674			56.522 ± 37.830			0.585
	2 Months Before	2 Months After	P	2 Months Before	2 Months After	P	–
FT3 (pmol/L)	5.295 (4.490,7.540)	4.765 (4.110,5.790)	0.000	4.770 (4.210,5.810)	4.950 (4.490,5.750)	0.756	–
FT4 (pmol/L)	16.490 (13.400,21.678)	15.765 (12.913,19.380)	0.000	16.050 (13.320,19.410)	16.740 (14.380,20.120)	0.246	–
TSH (mIU/L)	0.134 (0.005,1.758)	0.524 (0.006,2.170)	0.308	0.815 (0.017,2.850)	1.300 (0.035,3.170)	0.419	–
TRAB (IU/L)	6.300 (3.075,13.145)	4.455 (2.313,9.893)	0.000	4.450 (2.260,10.930)	4.470 (2.410,10.340)	0.237	–
TRAB Change (IU/L)	1.290 (0.440,2.770)			0.060 (-0.600,1.010)			0.000

Data are presented as mean*±*standard error (x*±*s) or median (interquartile range). Categorical outcomes were shown as absolute and relative prevalence of complications (%). FT3 free triiodothyronine 3, FT4 free thyroxine 4, TSH thyroid-stimulating hormone, TRAB thyrotropin receptor antibody.

### The Changes in Serum TRAB Levels Decreased Before Vaccination and Increased After Vaccination

To detect changes in serum thyroid function and TRAB levels at different time points before and after vaccination, we compared thyroid function and TRAB levels at 3 months, 1 month before and 1 month, 3 months after the first dose of vaccine. The baseline clinical data of the groups were analyzed statistically to exclude other influencing factors after vaccination for GD. No significant differences were observed between the groups in terms of sex, age, medication, and other clinical characteristics. The ANOVA analysis showed that there was a statistically significant difference in TRAB among the groups (p=0.019). The change trend of TRAB decreased before vaccination (5.880IU/L vs. 4.275IU/L) (p=0.009) and increased after vaccination (4.345IU/L vs. 4.475IU/L) (p=0.509). The change trends of FT3 and FT4 were both similar to those of TRAB, which decreased before vaccination (FT3: 5.220 pmol/L vs. 4.905 pmol/L, p=0.002; FT4: 16.415 pmol/L vs. 16.050 pmol/L, p=0.164) and increased after vaccination (FT3: 4.860 pmol/L vs. 4.990 pmol/L, p=0.247; FT4: 16.375 pmol/L vs. 16.840 pmol/L, p=0.271). The difference was that TSH levels continued to rise (0.218 mIU/L vs. 0.548 mIU/L vs. 0.817 mIU/L vs. 1.070 mIU/L) (p=0.000) (**Table 2**).

**Table 2:** Variation in thyroid function at different time points before and after vaccination.

	Pre-vaccination		Post-vaccination		P	Post Hoc
	3 Months Before (n = 280)	1 Month Before (n = 294)	1 Month After (n = 306)	3 Months After (n = 250)		
Gender (female%)	79.286	76.871	77.124	76.400	0.857	–
Age (year)	40.181 ±10.913	40.252 ±10.838	40.101 ±10.886	39.606±10.678	0.904	–
Duration (month)	11.150 (4.467,21.050)	13.917 (6.508,23.075)	15.283 (8.500,25.600)	18.367 (10.292,29.233)	0.000	–
Thiamazole (mg)	15.384±4.667	15.145±4.901	15.204±4.852	15.350±4.476	0.955	–
Letrox (ug)	53.214 ±37.309	52.976 ±38.665	51.797±38.476	53.200±37.555	0.968	–
FT3 (pmol/L)	5.220 (4.468,7.108)	4.905 (4.220,6.000)	4.860 (4.268,5.950)	4.990 (4.485,5.878)	0.006	0.002^a^ 0.997^b^ 0.247^c^
FT4 (pmol/L)	16.415 (13.313,21.478)	16.050 (13.300,19.413)	16.375 (13.410,20.118)	16.840 (14.465,19.915)	0.361	0.164^a^ 0.999^b^ 0.271^c^
TSH (mIU/L)	0.218 (0.005,2.018)	0.548 (0.006,2.283)	0.817 (0.012,2.595)	1.070 (0.017,3.133)	0.000	0.169^a^ 0.047^b^ 0.318^c^
TRAB (IU/L)	5.880 (2.708,13.070)	4.275 (2.203,10.310)	4.345 (2.288,10.110)	4.475 (2.453,9.853)	0.019	0.009^a^ 0.865^b^ 0.509^c^

Data are presented as mean*±*standard error (x*±*s) or median (interquartile range). Categorical outcomes were shown as absolute and relative prevalence of complications (%). FT3 free triiodothyronine 3, FT4 free thyroxine 4, TSH thyroid-stimulating hormone, TRAB thyrotropin receptor antibody.

a 3 Months Before versus 1 Month Before.

b 1 Month Before versus 1 Month After.

c 1 Month After versus 3 Months After.

### Baseline Characteristics of GD in Prospective Study

To further investigate the relationship between vaccination and serum TRAB levels, 173 GD who had received inactivated SARS-COV-2 vaccines were enrolled in a prospective study to compare the dynamic changes of serum TRAB levels before and at 3 and 1 months after vaccination. The baseline characteristics and clinical parameters of GD in this prospective study are summarized in **Table 3**. The mean age of the subjects in this study was 39.168 years, and 73.988% were women. The subjects had a median disease duration of 16.333 months, and their medication was MMI 14.957 mg and LT-4 58.960 µg on average. The baseline median serum FT3, FT4, TSH, and TRAB levels were 5.230 pmol/L, 15.930 pmol/L, 0.375 mIU/L, and 5.450 IU/L, respectively.

**Table 3:** Demographic and clinical characteristics of enrolled patients (n = 173).

Gender (female%)	73.988
Age (year)	39.168±10.713
Duration (month)	16.333 (9.617,26.309)
Thiamazole (mg)	14.957±5.293
Letrox (ug)	58.960±37.246
FT3 (pmol/L)	5.230 (4.500,6.570)
FT4 (pmol/L)	15.930 (13.530,20.300)
TSH (mIU/L)	0.375 (0.005,2.020)
TRAB (IU/L)	5.450 (2.555,11.350)

Data are presented as mean*±*standard error (x*±*s) or median (interquartile range). Categorical outcomes were shown as absolute and relative prevalence of complications(%). FT3 free triiodothyronine 3, FT4 free thyroxine 4, TSH thyroid-stimulating hormone, TRAB thyrotropin receptor antibody.

### Serum TRAB Levels Decreased Slightly After Vaccination and Showed an Upward Trend

**Figure 3:**
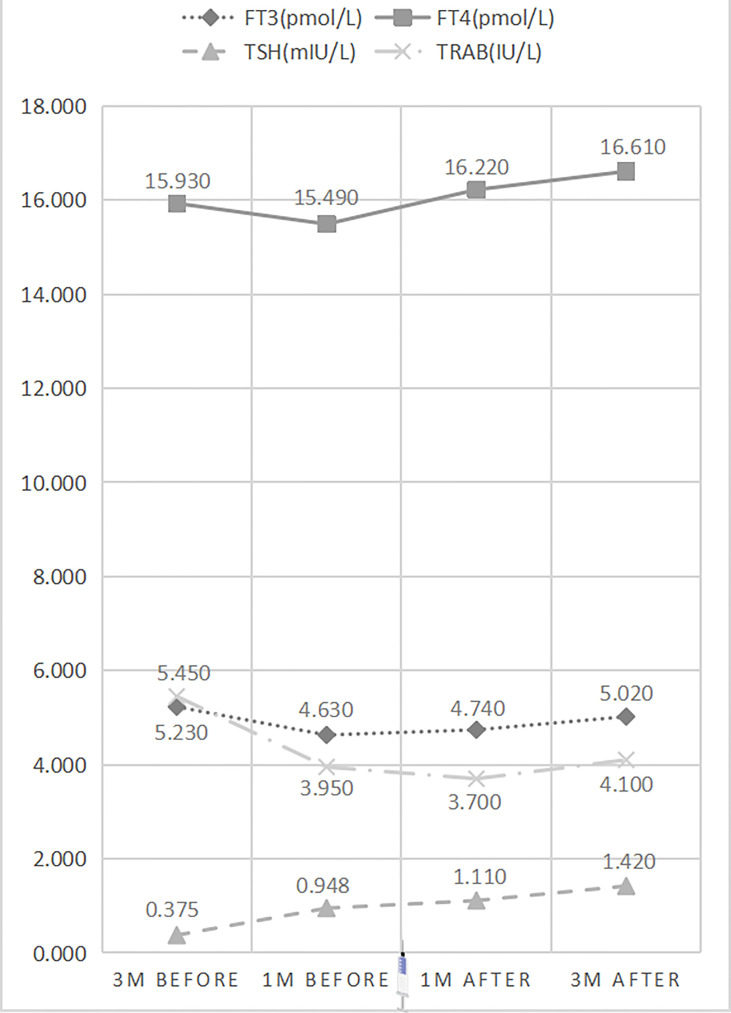
Line charts of variation in thyroid function of prospective subjects.

Repeated measures ANOVA indicated significant differences in serum FT3 (p=0.000), FT4 (p=0.000), TSH (p=0.000), and TRAB (p=0.000) levels at different time points before and after vaccination. Serum TRAB levels showed dynamic changes that decreased significantly at 1 month before vaccination (5.450IU/L vs. 3.950 IU/L) (p=0.000), no significant differences at 1 month after vaccination (3.95 0IU/L vs. 3.700 IU/L) (p=0.583), and a slight change and reflected an upward trend at 3 months after vaccination (3.700 IU/L vs. 4.100 IU/L) (p=0.034) (**Table 4** and **Figure 3**). Serum FT3 and FT4 levels showed similar trends to serum TRAB levels before and after vaccination. However, the slight differences were that FT3 and FT4 showed an upward trend at 1 month after vaccination, although there was no difference in the change of FT3 (4.630pmol/L vs. 4.740 pmol/L) (p=0.095) and FT4 (15.490 pmol/L vs. 16.220 pmol/L) (p=0.068) 1 month before and after vaccination, and the upward trend was more significant 3 months after vaccination (FT3: 4.740 pmol/L vs. 5.020 pmol/L, p=0.001; FT4: 16.220 pmol/L vs. 16.610 pmol/L, p=0.012). Instead, serum TSH levels showed a continuous upward trend over time (0.375 mIU/L vs. 0.948 mIU/L vs. 1.110 mIU/L vs. 1.420 mIU/L) (p=0.000).

**Table 4:** Variation in thyroid function of prospective subjects (n = 173).

	Pre-vaccination		Post-vaccination		P	Pairwise Comparison
	3 Months Before	1 Month Before	1 Month After	3 Months After		
FT3 (pmol/L)	5.230 (4.500,6.570)	4.630 (4.095,5.515)	4.740 (4.235,5.760)	5.020 (4.530,5.735)	0.000	0.000^a^ 0.095^b^ 0.001^c^
FT4 (pmol/L)	15.930 (13.530,20.300)	15.490 (12.910,18.760)	16.220 (13.540,19.145)	16.610 (14.660,19.400)	0.000	0.006^a^ 0.068^b^ 0.012^c^
TSH (mIU/L)	0.375 (0.005,2.020)	0.948 (0.017,2.700)	1.110 (0.042,2.765)	1.420 (0.204,3.225)	0.000	0.002^a^ 0.064^b^ 0.138^c^
TRAB (IU/L)	5.450 (2.555,11.350)	3.950 (2.080,8.780)	3.700 (2.000,7.835)	4.100 (2.360,8.965)	0.000	0.000^a^ 0.583^b^ 0.034^c^

Data are presented as median (interquartile range). Categorical outcomes were shown as absolute and relative prevalence of complications (%). FT3 free triiodothyronine 3, FT4 free thyroxine 4, TSH thyroid-stimulating hormone, TRAB thyrotropin receptor antibody.

a 3 Months Before versus 1 Month Before.

b 1 Month Before versus 1 Month After.

c 1 Month After versus 3 Months After.

## Discussion

In the present study, we combined retrospective and prospective studies to investigate the effect of inactivated SARS-CoV-2 vaccines on TRAB in patients with GD. The results of inter-group comparison and repeated measures ANOVA indicated that serum TRAB levels decreased less after inactivated SARS-CoV-2 vaccination and showed an upward trend. Similarly, the serum FT3 and FT4 levels increased after vaccination. To the best of our knowledge, this is the first comparative assessment of serum TRAB levels with inactivated SARS-CoV-2 vaccines in GD since the COVID-19 outbreak.

SARS-CoV-2 is spreading rapidly worldwide with high numbers of confirmed cases and fatality rates and limited treatment options. Widespread vaccination against COVID-19 is a crucial tool to control the pandemic. In China, most citizens are vaccinated with inactivated SARS-CoV-2. Inactivated vaccines are a mature technology with highly efficient proliferation and high genetic stability and are widely used for the prevention and control of emerging infectious diseases (11). Inactivated SARS-CoV-2 vaccines are made by taking live viral samples from multiple patients and replicating them in Vero cells of the African green monkey cell line, which is susceptible to infection (12). Then, the strain with the best proliferation and lowest mutagenesis rate is isolated, further proliferated, inactivated, and absorbed onto aluminum hydroxide to generate (13). In preclinical studies, inactivated SARS-CoV-2 vaccines provided complete protection against SARS-CoV-2 infection by triggering effective humoral immune responses and inducing SARS-CoV-2-specific neutralizing antibodies in serrated animals and nonhuman primates (14). Furthermore, vaccines have been found to elicit a rapid humoral response in healthy individuals to be tolerable and immunogenic (15).

Adjuvants are compounds added to vaccines to enhance immunogenicity, which could lead to practical advantages, including dose-sparing and inducing a more rapid, strong, and long-lasting immune response (16, 17). Aluminum compounds are the most widely used adjuvants for human vaccines. Aluminum traps soluble antigens, interacts with dendritic cells, enhances antigen presentation and complement and eosinophil activation, promotes an influx of neutrophils, enhances the secretion of pro-inflammatory cytokines and chemokines, and reduces immunopathology, elevating protective immunity levels to the threat of homologous viruses (18). While everything has two sides, the more effective it is, the higher is the risk. However, adjuvants are not completely free of side effects. In genetically predisposed individuals, adjuvants may induce ASIA by disrupting the host’s immunological balance through molecular simulations, triggering polyclonal activation of B lymphocytes, or other similar etiological mechanisms (19). Adjuvants can trigger generalized autoimmune reactions, resulting in multiple autoantibodies, and contribute to the development of autoimmune diseases including AITD, rheumatoid arthritis (RA), systemic lupus erythematosus (SLE), Sjogren’s syndrome (SS), Guillain Barre syndrome (GBS), and multiple sclerosis (18).

GD is the most common cause of hyperthyroidism. Under normal conditions, TSH receptors (TSHRs) located on the surface of thyroid cells bind to TSH, which activates adenylate cyclase and phosphoinositol-dependent signaling pathways to produce thyroid hormones (20). Hyperthyroidism is caused by the growth and reproduction of thyroid cells and persistent and uncontrolled thyroid stimulation resulting from the interaction of TRAB with TSHR in genetically predisposed individuals with GD. The pathogenesis of GD involves the destruction of thyroid immune tolerance, with the most pathogenic antibody being TRAB, and the immune mechanism is complex. A large amount of evidence has shown that the active phase of GD is associated with an immune prevalence of the Th1 immune response, whereas the inactive or later phases of GD are associated with a switch from Th1 to Th2 immune prevalence (21). It has been speculated that the mechanism of immune tolerance disruption is the maladjustment of autoreactive B cells that switch to plasma cells that produce pathogenic immunoglobulin G (20). Moreover, increasing the stimulating effect of Th2 cells on B cells promotes the production of more TRABs, which is considered another mechanism of GD occurrence (20).

TRABs were explored as an entry point of this study as an important indicator to evaluate treatment efficacy and recurrence. We conducted a retrospective study to observe the differences in TRAB trends between pre-vaccination and post-vaccination. Before vaccination, serum thyroid function and TRAB levels decreased significantly at intervals of 2 months under standard treatment, indicating an effective treatment. In contrast to pre-vaccination, no significant differences were found in serum thyroid function and TRAB levels post-vaccination, which indicated that there was no improvement in thyroid function and TRAB after vaccination under the same treatment regimen. Simultaneously, we compared serum TRAB levels at specified time points before and after vaccination. The results showed that the TRAB increased after vaccination, which also supports the previous prediction. The differences in the duration of the retrospective study were due to the overlap among subgroups of GD enrolled during the research, with the course of disease apparently longer post-vaccination than before. Generally, serum TRAB levels should decline over time with standard treatment, which was not observed in our study. However, an unexpected increase in TRAB levels was observed after vaccination. The expected trend of TRAB titer is to decrease before vaccination, which contrasts with the TRAB titer plateau immediately after vaccination (+1-month time point) and the subsequent inverted and unexpected trend of TRAB increase at +3-months time-point after vaccination. To further explore the dynamic changes in TRAB before and after vaccination, we expanded a prospective study that measured the levels of serum thyroid function and TRAB at 3 and 1 months before and after vaccination in GD and then performed repeated measurement ANOVA. Lifestyle and treatment of the enrolled subjects were unaltered during the study. Consistent with the results of this retrospective study, there were significant differences in serum FT3, FT4, and TRAB levels at different time points before and after vaccination. Serum TRAB levels showed dynamic changes that decreased significantly at 1 month before vaccination, showed no significant differences at 1 month after vaccination, and changed slightly and reflected an upward trend at 3 months after vaccination. Serum FT3 and FT4 levels showed trends similar to those of serum TRAB levels before and after vaccination, but the nuances were that their upward trend moved forward and appeared 1 month after vaccination. Surprisingly, the changes in TSH after vaccination 1-3 months were inconsistent with those in FT3 and FT4, showing a continuous upward trend. We speculate that this phenomenon may be related to the sensitivity of TSH, which usually changes earlier than FT3 and FT4 levels. It may be that thyroid hormone is about to decline at the time point, but we have not captured their decreased levels due to the short follow-up time.

Based on the results obtained in both retrospective and prospective studies, we concluded that serum TRAB levels decreased less after vaccination and showed an upward trend. Thinking along the lines above, the association between inactivated SARS-CoV-2 vaccines and serum TRAB levels may be related to humoral immunity. After vaccination, antibodies were generated through humoral immunity, which could stimulate B cells and promote the synthesis of TRABs, changing the original declining trend of TRABs. Another explanation may be that adjuvants added to inactivated vaccines may disrupt the host immune balance and stimulate B cell cloning, affecting the original trend of TRAB.

Our study has two limitations. First, the current study was conducted in a single-center cohort of patients, with the possibility of selection bias. Second, the short follow-up period in our study resulted in unclear trends in serum TRAB levels 3 months after vaccination. The follow-up period should be extended to validate the time point of the peak of its increase.

The data presented here demonstrate that inactivated SARS-CoV-2 vaccines may affect TRAB trends in GD patients. Nevertheless, we must emphasize that the initial goal of vaccination is to protect the population from infection and reduce infection and mortality, and inactivated SARS-CoV-2 vaccines are not contraindicated in patients with GD (22). COVID-19 has led to millions of disabilities and deaths worldwide, especially in men, the elderly, and those with previous health problems; therefore, we believe that the risks of COVID-19 outweigh the minor risks of the vaccine in these populations. However, autoimmune diseases, especially GD, predominantly affect young women, who have a significantly reduced risk of severe Covid-19 disease. Thus, a careful analysis of the risk/benefit ratio should be continuously applied and revised according to the new scientific data that are produced daily. The results of this study provide evidence for clinical management and clinicians should be aware that TRAB levels may stop declining after vaccination.

## Conclusion

Taken together, based on retrospective and prospective studies, the data presented here demonstrate that serum TRAB levels decreased less after inactivated SARS-CoV-2 vaccination and showed an upward trend, and FT3 and FT4 were consistent with it. This may be related to humoral immunity induced by vaccination. This finding suggests that humoral immunity induced by inactivated SARS-CoV-2 vaccine may affect autoimmunity. The advantages and disadvantages of vaccination should be weighed according to the applicable population. Clinicians should be aware that TRAB levels may stop declining following vaccination.

## Data Availability Statement

The raw data supporting the conclusions of this article will be made available by the authors, without undue reservation.

## Ethics Statement

The studies involving human participants were reviewed and approved by The Second affiliated Hospital of Fujian Medical University. The patients/participants provided their written informed consent to participate in this study.

## Author Contributions

All authors planned the concept of this report and wrote and revised the final manuscript. All authors contributed to the article and approved the submitted version.

## Funding

This work was supported by Science and Technology Project of Fujian Provincial Department (2019J01166), Innovative Medical Research Project of Fujian Province (2018-CX-33), and High-Level Talent Program of Science and Technology Project of Quanzhou (2018C044R).

## Conflict of Interest

The authors declare that the research was conducted in the absence of any commercial or financial relationships that could be construed as a potential conflict of interest.

## Publisher’s Note

All claims expressed in this article are solely those of the authors and do not necessarily represent those of their affiliated organizations, or those of the publisher, the editors and the reviewers. Any product that may be evaluated in this article, or claim that may be made by its manufacturer, is not guaranteed or endorsed by the publisher.
